# Seventy-year retained calcified *Schistosoma japonicum* ova incidentally found in a rectal cancer resection specimen

**DOI:** 10.1016/j.ijregi.2025.100704

**Published:** 2025-07-10

**Authors:** Emiri Muranaka, Koya Yokoyama, Makio Kawakami, Ryota Hase

**Affiliations:** 1 Japanese Red Cross Narita Hospital, Department of Infectious Diseases, 90-1 Iida-cho, Narita, Chiba 286-8523, Japan; 2 Japanese Red Cross Narita Hospital, Department of Surgery, 90-1 Iida-cho, Narita, Chiba 286-8523, Japan; 3 Japanese Red Cross Narita Hospital, Department of Pathology, 90-1 Iida-cho, Narita, Chiba 286-8523, Japan

**Keywords:** Schistosoma japonicum, Rectal cancer, Calcified ova, Japan, Chronic schistosomiasis

An 82-year-old Japanese man underwent low anterior resection for well-differentiated tubular adenocarcinoma (Stage IIIa). Histopathological examination of the resected specimen (Figure 1, hematoxylin and eosin stain) revealed approximately 20–25 oval calcified structures measuring 85–95 × 60–70 µm (mean: 90 × 65 µm) in the submucosal layer, consistent with *Schistosoma japonicum* ova. The ova were visualized as black calcified structures using Kossa stain (Figure 2). Due to the calcified and degenerated nature of the ova after seven decades, the characteristic lateral spine of *Schistosoma japonicum* was not clearly visible. The surrounding tissue showed no significant inflammatory infiltration, fibrosis, or granulomatous changes suggestive of chronic inflammation. The patient had no evidence of liver fibrosis or splenomegaly.

**Figure 1:**
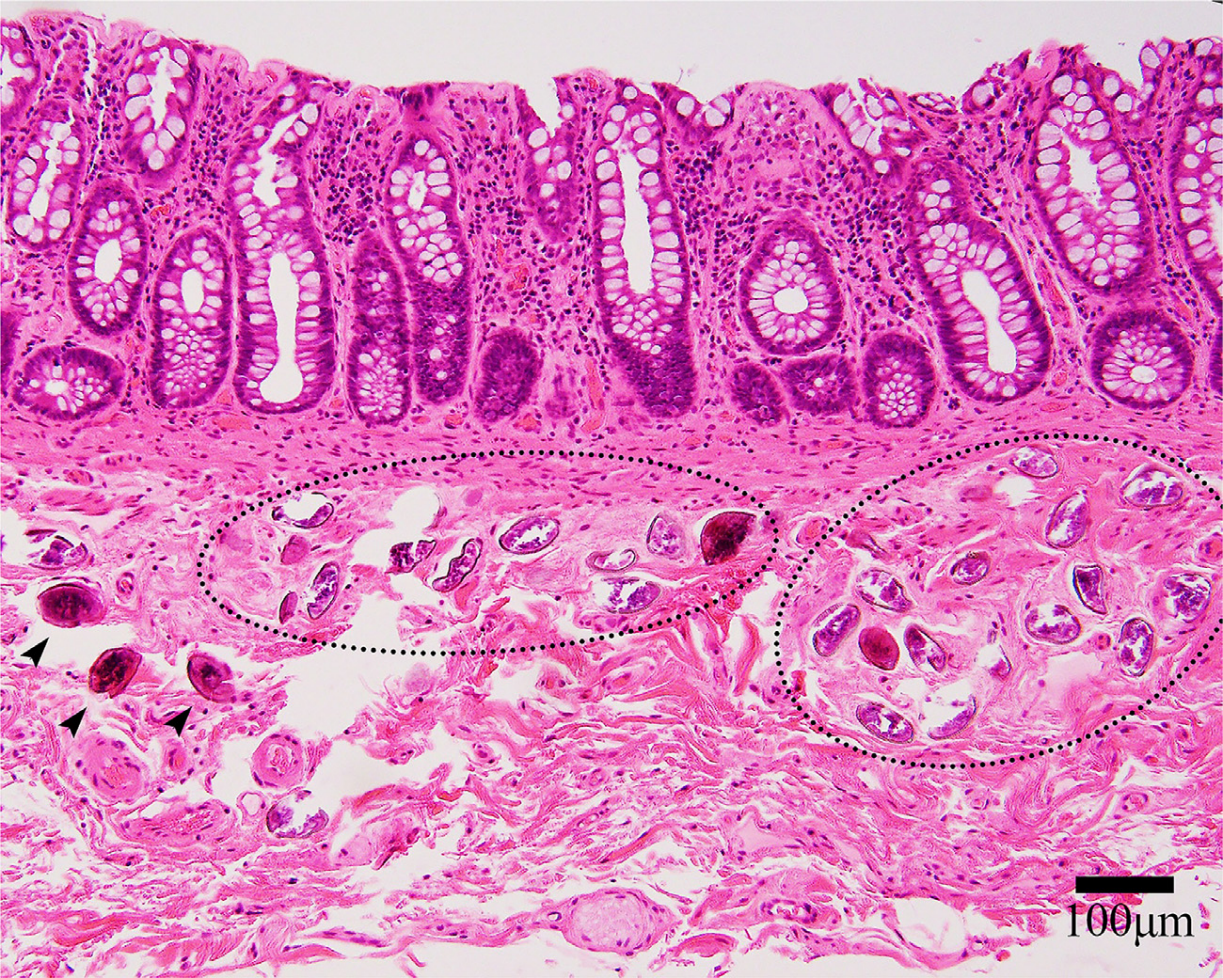
Histopathological examination of rectal tissue (×200 magnification, hematoxylin & eosin stain) showing normal colonic glandular structures in the upper mucosal layer with multiple oval-shaped calcified *Schistosoma japonicum* ova (arrows) visible in the submucosal layer. The ova measure approximately 85-95 × 60-70 µm and appear as oval structures with basophilic granular contents. Note the absence of inflammatory infiltration around the calcified ova. Scale bar = 100 µm.

**Figure 2:**
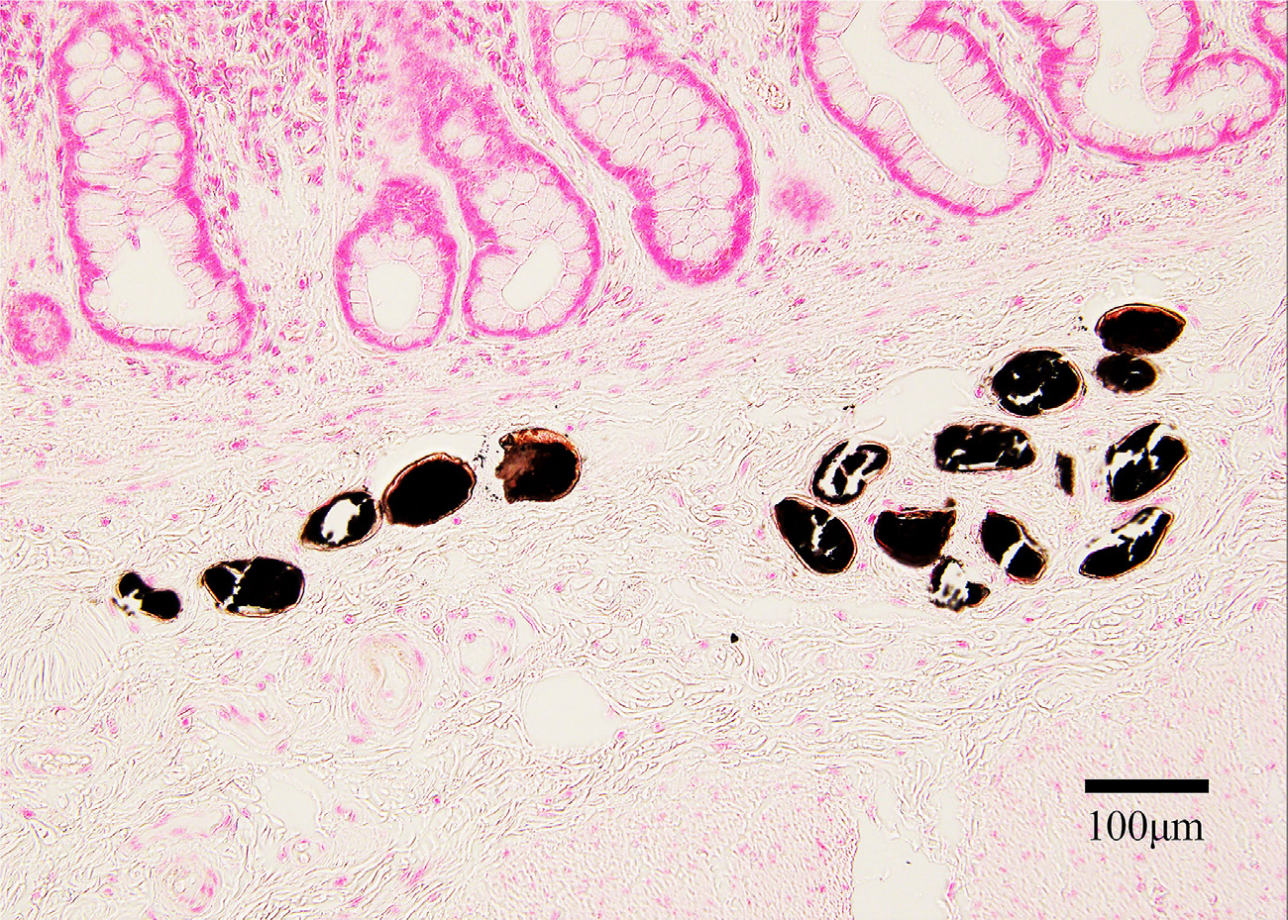
von Kossa staining of the same tissue section (×200 magnification) highlighting multiple calcified *Schistosoma japonicum* ova as black deposits, confirming the calcified nature of these 70-year-old parasitic structures in the rectal submucosa. Scale bar = 100 µm.

The patient had spent his childhood in the Chikugo River basin of Fukuoka Prefecture, a former endemic area for Schistosoma japonicum in Japan [1]. He reported having received mass deworming treatment 70 years prior. Notably, his mother had also undergone rectal resection for rectal cancer, with similar ovarian findings in the specimen. Given that the lifespan of schistosomes in the human body is approximately 3–5 years [2], this represents a case of chronic schistosomiasis with 70-year retained calcified ova, and anthelmintic treatment was deemed unnecessary.

While the clinical significance of long-term retained calcified ova remains unclear, the International Agency for Research on Cancer classifies *S. japonicum* as Group 2B (possibly carcinogenic) [3], and epidemiological studies from endemic areas have reported increased colorectal cancer rates [4]. In the present case, although a few calcified ova were observed near the cancer tissue, similar ova were equally distributed throughout the normal colonic mucosa, making it difficult to establish a clear causal relationship between chronic schistosomiasis and cancer development.

Through extensive environmental control measures targeting the intermediate snail host (*Oncomelania hupensis nosophora*), Japan successfully eliminated schistosomiasis by 1977. These control efforts were particularly intensive in endemic areas such as the Chikugo River basin, where comprehensive approaches included concrete lining of irrigation canals, land reclamation, and the systematic application of molluscicides. The Japanese government's coordinated public health interventions, combined with mass screening and treatment programs, led to the complete interruption of transmission [5].

This case highlights the importance of considering schistosomiasis in the differential diagnosis of patients with a history of exposure, even in non-endemic regions, as ova can persist in tissues long after the death of adult worms.

## Declarations of competing interest

The authors have no competing interests to declare.
